# Who Is Listening? Spokesperson Effect on Communicating Social and Physical Distancing Measures During the COVID-19 Pandemic

**DOI:** 10.3389/fpsyg.2020.564434

**Published:** 2021-01-12

**Authors:** Ahmad Abu-Akel, Andreas Spitz, Robert West

**Affiliations:** ^1^Institute of Psychology, University of Lausanne, Lausanne, Switzerland; ^2^Institute of Computer and Communication Sciences, École Polytechnique Fédérale de Lausanne, Lausanne, Switzerland

**Keywords:** COVID-19, pandemic (COVID-19), public health messaging, spokesperson, effective communication, celebrity

## Abstract

Effective communication during a pandemic, such as the current COVID-19 crisis, can save lives. At the present time, social and physical distancing measures are the lead strategy in combating the spread of COVID-19. In this study, a survey was administered to 705 adults from Switzerland about their support and practice of social distancing measures to examine if their responses depended on (1) whether these measures were supported by a government official or an internationally recognized celebrity as a spokesperson, (2) whether this spokesperson was liked, and (3) the respondent’s age. We also considered several attitudinal and demographic variables that may influence the degree to which people support and comply with social distancing measures. We found that the government official was more effective in eliciting responses supportive of social distancing, particularly as manifested in the stated current compliance with social distancing measures. The effect was substantially stronger among older respondents, although these respondents expressed a lower risk perception. Although there was a general trend for greater endorsement of the social distancing measures among participants who liked the spokesperson, this was non-significant. In addition, respondents’ greater support and compliance was positively associated with (1) higher concern for the current situation, (2) higher concern for the well-being of others, and (3) greater belief that others were practicing social distancing, and negatively with (4) greater self-reported mobility. Current compliance correlated negatively with (5) household size. Since different parts of the population appear to have different perceptions of risk and crisis, our preliminary results suggest that different spokespersons may be needed for different segments of the population, and particularly for younger and older populations. The development of evidence-based knowledge is required to further identify who would be the most effective spokesperson, and in particular to groups with low risk perception and low compliance.

## Introduction

In an effort to avert the spread of the coronavirus disease 19 (COVID-19), a commonly given instruction is to practice social and physical distancing, generally defined as deliberately keeping a distance of at least 2 m (6 feet) from other people. To enforce this measure, people are advised, instructed, or even mandated to cancel sports events, cruises, festivals, and other gatherings; cancel or postpone conferences and large meetings; work from home instead of at the office; close schools, universities, and daycare centers; and visit loved ones through the use of electronic devices instead of in person (Gostin et al., 2020; JHU, 2020; Maragakis, 2020). This survey-based study aims to contribute to the development of evidence-based knowledge to improve our communication efforts in responding to unprecedented health crises such as the current COVID-19 crisis. Specifically, the current study sought to investigate the influence of the messenger (spokesperson) on inducing support for, and compliance with social distancing measures.

In times of pandemics, public health messaging with the affected population in a coordinated, effective, and credible way is considered a key factor in controlling the spread of the disease. Beyond the content of the message, the person who communicates the message–the spokesperson–is one of the most important factors that could determine the effectiveness of the message, particularly during times of heightened uncertainty as during emerging infectious diseases (Vaughan and Tinker, 2009; Lyu et al., 2013). During the current COVID-19 crisis, in addition to designating government official spokespersons, governments have also resorted to enlisting celebrities to bring heightened awareness about the pandemic (Swiss Federal Office of Public Health, 2020), and more recently to persuade people to take the coronavirus vaccine (Campbell, 2020). Enlisting celebrities may seem a reasonable strategy, given evidence suggesting that celebrities who are viewed favorably consistently have positive effects on people’s opinions, attitudes, and behaviors (Jackson and Darrow, 2005; Jackson, 2018), perhaps through a pseudo-personal, one-way rapport (Basil, 1996). However, little is known about the effect of celebrity spokespersons in times of crises. In a rare study that investigated the effect of a government official compared to a celebrity spokesperson during *hypothetical* crises (humanitarian and security), support for intervention or increased interest in the crisis were lower when the cue came from the celebrity rather than the government official (Frizzell, 2011). The extent to which this effect manifests in *real* crises is greatly understudied (Belt, 2011).

In addition, studies investigating responses during the early stages of prior pandemics have identified a number of important demographic, attitudinal, and psychological factors that could influence compliance (DiGiovanni et al., 2004; Bish and Michie, 2010; Reddy and Gupta, 2020). With respect to demographic factors, evidence from previous and the current COVID-19 pandemic suggest that age is a key factor, with young adults are likely to be least compliant (Bish and Michie, 2010; Barari et al., 2020; Everett et al., 2020; Pfattheicher et al., 2020; YouGov, 2020). For example, preliminary findings from Italy suggest that, while public messaging is generally being adhered to, this is true to a lesser degree among young adults (Barari et al., 2020). Similarly, while 76% of United States adults (at least 18 years old) reported that they were practicing social distancing, this was reported only in 67% of young adults between 18 and 34 years of age (YouGov, 2020). Moreover, it has been reported that older people felt more responsible for preventing the spread of the disease and expressed stronger intentions to practice social distancing measures such as avoiding gatherings and staying in self-isolation (Everett et al., 2020). Similarly, attitudinal factors (e.g., perceived health status, attitudes toward public health, and government officials) have been shown to influence the degree to which people support, and comply with, social distancing measures (Bish and Michie, 2010; Barari et al., 2020; Everett et al., 2020; Pfattheicher et al., 2020; YouGov, 2020). For example, greater trust in authorities has been associated with adopting protective behaviors (Bish and Michie, 2010). Psychological factors such as risk perception and concern for others have also been shown to affect compliance (Bish and Michie, 2010; Pfattheicher et al., 2020; Wise et al., 2020). For example, concern for others (empathy) has been associated with the motivation to adhere to physical distancing and to wearing face masks (Pfattheicher et al., 2020), and the willingness to restrict one’s own mobility to “flatten the curve” was particularly high when the motivation was to protect vulnerable others (Betsch, 2020). The protection motivation theory (PMT) (Maddux and Rogers, 1983) suggests that these factors are guided by threat appraisal processes–which assess the severity and seriousness of the situation/health information and coping appraisal processes–which assess the cost-benefit ratio of the response to the situation/health information (see also Schimmenti et al., 2020).

However, research on compliance with public health messaging during health crises has primarily focused on how these factors might relate to the content of the message, and considerably less so to the messenger (Nyhan et al., 2014; Bavel et al., 2020). This is particularly important given research showing that the message content alone may have no or even counterproductive effect on compliance with recommendations regarding diseases of great risk to public health (Nyhan et al., 2014; Nyhan and Reifler, 2015). The current study was thus conducted with three main goals in mind. Our first main goal was to assess, among adults in Switzerland, (1) whether self-reported support for, and current and future compliance with, social distancing measures depended on the spokesperson stated to have supported these measures (Swiss President Simonetta Sommaruga or celebrity actor Tom Hanks), and (2) whether these differences depended on the respondent’s sentiment toward the spokesperson, that is, on the extent to which the spokesperson is liked. We predicted that respondents would express more favorable responses to social distancing measures when the spokesperson is a liked celebrity.

Our second main goal was to examine whether support for, and compliance with social distancing measures is age-dependent. We predicted that, while the younger respondents would express lesser support and practice of social distancing measures, the celebrity would have a greater effect on them than the government official. In addition, our third goal was to examine the potential association of several attitudinal and demographic factors with engagement in social distancing (see section “Materials and Methods” for details) (Bish and Michie, 2010; Barari et al., 2020; Everett et al., 2020; Pfattheicher et al., 2020; YouGov, 2020), and whether the effects of spokesperson and age can be observed when adjusting for these factors.

## Materials and Methods

### Participants

Two online surveys (see Supplementary Appendix) were randomly assigned to 705 respondents (see Table 1 demographic details, see section “Preliminary Analysis”). In one survey, social distancing was supported by Simonetta Sommaruga (sitting President of the Swiss Confederation), and in the other survey, social distancing was supported by Tom Hanks (a celebrity actor). The two surveys were identical in all other respects. Respondents were recruited via a targeted ad campaign to users of Facebook and via a university online research platform. The Facebook ad consisted of rendered image of the virus, the sentence “Help us understand how the COVID-19 is affecting people’s lives in a 3-min survey,” and a link that redirected the respondent to one of two survey forms. The university online platform sent emails to registered university students, which randomly contained a link to one of the survey forms. Responses were digitally captured and downloaded for data processing at the end of the study period (see section “The Study in Context”). The study was conducted in compliance with the EPFL Human Research Ethics Committee guidelines.

**TABLE 1:** Demographic details of the overall and subsamples.

Sample variable	Overall sample (*N* = 705)	Facebook users (*N* = 447)	University students (*N* = 258)
Age (mean ± σ)	34.35 ± 16.46	42.02 ± 16.04	21.05 ± 3.94
**Gender (%)**			
Male	155(22%)	111(24.8%)	44(17.1%)
Female	548(77.7%)	335(74.9%)	213(82.6%)
Other	2(0.3%)	1(0.2%)	1(0.4%)
Employment (%)			
Employed	364(51.6%)	292(65.3%)	72(27.9%)
Unemployed	341(48.4%)	155(34.7%)	186(72.1%)
**Education (%)**			
No schooling	1(0.1%)	1(0.2%)	0(0.0%)
1–6 years	22(3.1%)	21(4.7%)	1(0.4%)
7–13 years	116(16.5%)	116(26.0%)	0(0.0%)
14–16 years	328(46.5%)	152(34.0%)	176(68.2%)
17–18 years	149(21.1%)	81(18.1%)	68(26.4%)
Over 18 years	89(12.6%)	76(17.0%)	13(5.0%)
Household size (mean ± σ)	3.08 ± 1.38	2.74 ± 1.32	3.68 ± 1.28
**Settlement size (%)**			
Village	223(31.6%)	154(34.5%)	69(26.7%)
Small Town	221(31.3%)	126(28.2%)	95(36.8%)
Town	148(21.0%)	90(20.1%)	58(22.5%)
City	105(14.9%)	71(15.9%)	34(13.2%)
Metropolitan	8(1.1%)	6(1.3%)	2(0.8%)

### Study Material

The survey (see Supplementary Appendix) elicited, on a 7-point Likert scale, responses that gauged the extent to which respondents (1) supported social distancing (*To what degree do you support social distancing as a valid measure in the current situation?*), (2) currently practiced social distancing (*To what degree are you currently practicing social distancing?*), and (3) intended to practice social distancing in the future (*To what degree do you see yourself practicing social distancing in the weeks to come?*). These three questions constitute the main outcome measures of the study. They were posed after an informative text block describing social distancing measures and a statement that these measures had been publicly supported by a randomly sampled one of the two spokespersons. The statement was accompanied by a portrait picture of the spokesperson. The two spokespersons were selected to, respectively, represent a source of official government instructions on social distancing (Simonetta Sommaruga) and an unofficial endorsement by an unaffiliated celebrity (Tom Hanks). To avoid spreading misinformation, we ensured that both speakers had actually previously issued public support of social distancing. Simonetta Sommaruga was chosen as the highest-ranking Swiss government official to have issued such support, while Tom Hanks was selected as a celebrity spokesperson who is well-liked, well-known across age groups and to an international audience (McDonald, 2013), and made headlines for his public endorsement of social distancing prior to the study and his coronavirus infection. The wording of the social distancing message was adapted from the definition by Johns Hopkins Medicine (JHU, 2020).

Participants were also asked whether they liked, disliked, were neutral toward, or did not know the spokesperson. In addition, the following demographic and attitudinal variables were collected: age, gender (male, female, other), employment status (employed and unemployed), years of education, household size, settlement size (village, small town, town, city, and metropolitan area), general health (on a 5-point Likert scale from very good to very bad), and perceived fraction of population infected by coronavirus (in 10% increments on a 100% scale). In addition, we asked the respondents to indicate on a 7-point Likert scale their level of concern about COVID-19, concern for the well-being of others, perception of others’ practice of social distancing, religiosity, liberty of movement (henceforth, mobility), satisfaction with the government’s efforts to combat COVID-19, and perception of the government’s concern for public health versus the economy.

### The Study in Context

**FIGURE 1:**
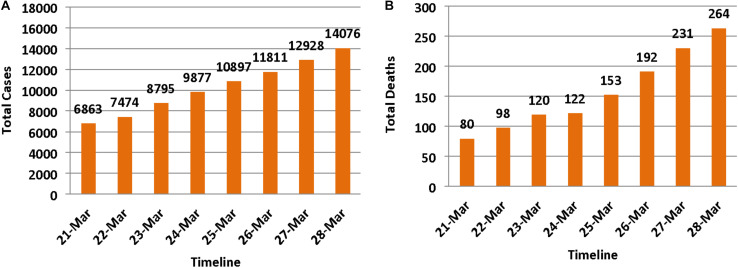
Total cumulative number of confirmed COVID-19 cases and deaths in Switzerland during the study period, 22–27 March, 2020 (Worldmeter, 2020). **(A)** The number of people who were infected with virus SARS-CoV-2, and **(B)** the number of COVID-19 related deaths. Numbers are likely to be much higher, particularly when, as of March 6, targeted testing strategy was the official policy in Switzerland (derbund.ch, 2020).

The survey was administered during the period of March 22–27, 2020, 6 days after the Swiss Federal Council had categorized the situation as *extraordinary* under the terms of the Epidemics Act (FOPH, 2020). From February 25, when the first case was confirmed in Switzerland (Thelocal.ch, 2020), a number of social distancing measures were progressively introduced by the Federal Council, which among other measures, included closing non-essential businesses on March 16 (6 days before the start of the survey), and limiting gatherings to a maximum of five persons on March 20 (2 days before the start of the survey) (FOPH, 2020). In addition, by the start of the survey, there were 7,474 confirmed cases, and 98 COVID-19 related deaths in Switzerland (see Figure 1 for total cumulative cases and deaths during the study period).

### Statistical Analyses

First, we computed Spearman’s correlation between the study variables. For the main analyses, we performed a series of multivariable regressions to examine the effect of the spokesperson on the responses to the three attitudinal questions about social distancing: (1) support, (2) current practice, and (3) future practice. Analyses were conducted while controlling for all demographic and attitudinal measures listed above. Analyses were performed in the entire sample and as a function of age group (see Supplementary Figure 1 and clustering details in the Supplementary Material). In addition, Kruskal–Wallis *H*-tests were used to compare young to old participants on all study measures. For gender differences, two respondents who indicated “Other” as their gender were excluded. For the regression analyses, 11 participants were excluded due to the small number of respondents in the following response categories: “Other” gender = 2, “no schooling” = 1, and living in a metropolitan area = 8.

To account for multiple testing, we applied false-discovery rate (FDR) correction (*q*-value = 0.05) (Benjamini and Hochberg, 1995). Effect sizes are reported in terms of Cohen’s *d* (mean difference divided by pooled standard deviation, reported in absolute values), partial eta-squared (η_*p*_^2^), and Cramer’s *V* as appropriate.

## Results

### Preliminary Analysis

**FIGURE 2:**
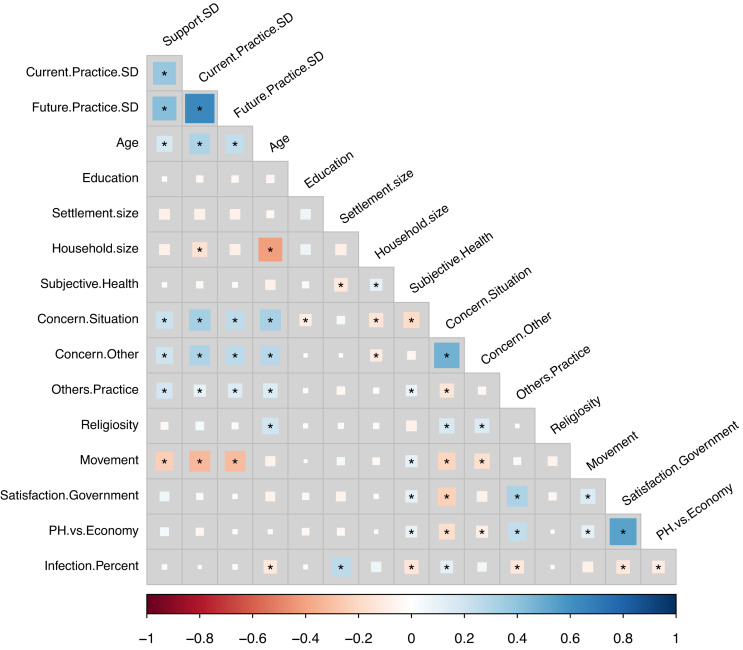
Spearman’s correlation matrix between the study variables. Square color and size, respectively, indicate direction and size of the correlation coefficients. Asterisks indicate significant correlation coefficients after false-discovery rate (FDR) correction for multiple testing. Figure was constructed with the R package *ggcorrplot*. SD, social distancing; PH, public health.

Demographics and details of the respondents are summarized in Table 1. About 98.3% of all respondents stated that they were aware of the social distancing measures at the time of the study. There were no statistically significant differences between employed and unemployed respondents on any of the three social distancing measures (all *H* < 0.89, *p* > 0.345), or between female and male respondents on current (*H* = 1.59, *p* = 0.207) or future practice (*H* = 0.97, *p* = 0.324). However, female respondents reported greater support for social distancing measures (*H* = 5.47, *p* = 0.019, *d* = 0.16). Spearman’s correlations (see Figure 2) revealed a significant positive associations between all three attitudinal measures of social distancing (support, current, and future practice) and the respondents’ age, concern for the current situation, concern for others, others’ practice of social distancing, as well as a significant negative association with movement (mobility). Furthermore, household size was significantly and negatively associated with the respondents’ degree of current practice of social distancing.

**FIGURE 3:**
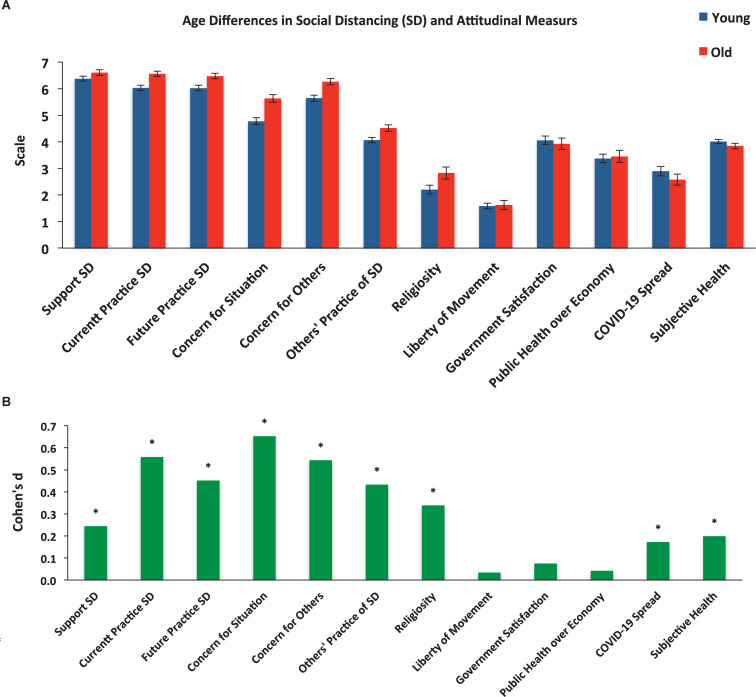
Differences in the social distancing and attitudinal measures between the younger and older groups. **(A)** Displays the means and 95% CI. **(B)** Displays Cohen’s *d* effect sizes (in absolute values). Asterisks denote false discovery rate-corrected significant effects (*q*-value = 0.05). SD, social distancing.

Moreover, given the bimodal structure of the age distribution of our sample (Supplementary Figure 1 and clustering details in the Supplementary Material), we compared differences between the younger (17–36 years of age) and older (37–80 years of age) groups on all the social distancing and attitudinal measures of the study. As can be seen in Figure 3, the scores reported by the older group were significantly higher for all questions, except for the perception of the spread of COVID-19 and the state of their general health, where the younger group reported higher scores. There was no difference between the age groups in movement (mobility), satisfaction with the government and the government’s prioritization of public health over the economy (see Supplementary Table 1 for details).

### Spokesperson Effect

**FIGURE 4:**
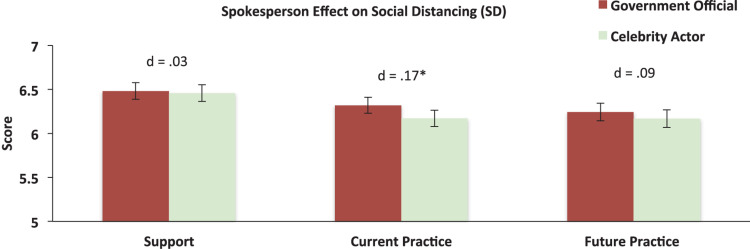
Spokesperson effect on the respondents’ support, current practice, and future practice of social distancing. Error bars represent 95% CIs; *d* = Cohen’s *d* effect size, *p < 0.05.

Accounting for all demographic and attitudinal variables, the multivariable regressions revealed that the government official had a small but significant effect on the reporting of current practice of social distancing measures [*F*(1,688) = 5.07, *p* = 0.025, Cohen’s *d* = 0.17]. We did not observe a statistically significant spokesperson effect for the respondents’ support or future practice of social distancing (see Figure 4).

Supplementary Tables 2–4 summarize the regression coefficients of the association of the demographic and attitudinal variables with each of the three social distancing outcome measures. For the support of social distancing (see Supplementary Table 2), parameter estimates revealed significant positive associations with the concern for others, concern for the situation, and others’ practice of social distancing, and significant negative associations with settlement size, religiosity, and mobility.

For current practice of social distancing (see Supplementary Table 3), parameter estimates revealed significant positive associations with age, concern for others, concern for the situation, others’ practice of social distancing, and with satisfaction from the government effort. Significant negative associations were observed for city size, mobility, and employment, where the employed reported lesser practice of social distancing measures than the unemployed.

For future practice of social distancing (see Supplementary Table 4), parameter estimates revealed significant positive associations with age, concern for others, concern for the situation, and others’ practice of social distancing, and a significant negative association with mobility.

### Effect of Spokesperson Likeability

Respondents indicated if they liked, disliked, were neutral toward, or did not know the spokesperson. Chi-squared analysis revealed significant differences in the distribution of the responses across the two spokespersons (*χ^2^* = 39.88, df = 3, *p* < 0.001, Cramer’s *V* = 0.24; see Table 2).

**TABLE 2:** Cross-tabulation of spokesperson likeability.

Spokesperson	Like	Neutral	Dislike	Don’t know
Government official (%)	87(25%)	151(44%)	11(3%)	97(28%)
Celebrity actor (%)	149(43%)	150(43%)	2(1%)	47(14%)

**FIGURE 5:**
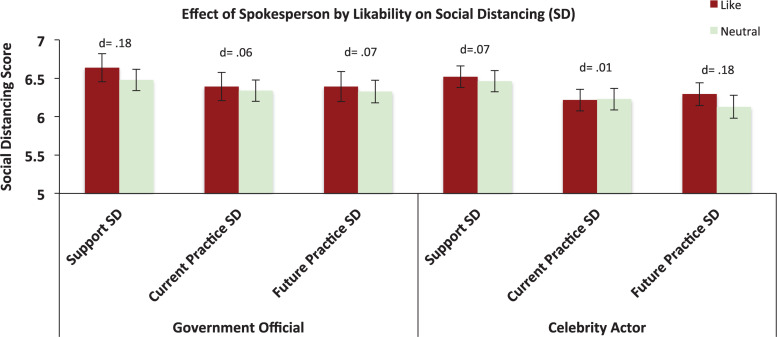
Effect of spokesperson likeability on the respondents’ support, current practice, and future practice of social distancing. Error bars represent 95% CIs; *d* = Cohen’s *d* effect size (in absolute values).

As can be seen from Table 1, overall only 1.87% of the respondents expressed dislike toward the spokespersons. Given this small number, the effect of the spokesperson’s likeability was only analyzed with respect to “like” versus “neutral.” Accordingly, we performed a series of multivariable regression analyses in which we also included the “Likability” factor and the “Likeability × Spokesperson” interaction term. Respondents who liked the spokesperson tended to report higher levels of support and practice of the social distancing measures, although these effects were not statistically significant (see Figure 5). Models details and parameter estimates are provided in the Supplementary Tables 5–7.

### Spokesperson Effect in Younger Versus Older Adults

**FIGURE 6:**
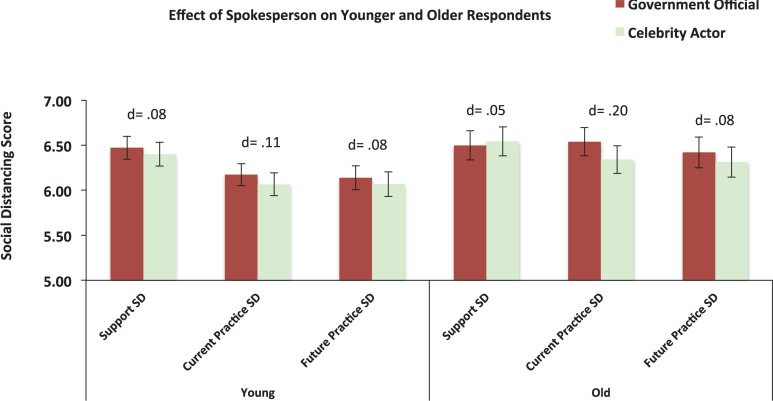
Spokesperson effect on the respondents’ support, current practice, and future practice of social distancing in the younger and older respondents. Error bars represent 95% CIs; *d* = Cohen’s *d effect size* (in absolute values).

We performed a series of multivariable regression analyses in which we also included the “Age Group” factor and the “Age Group × Spokesperson” interaction term (see Supplementary Tables 8–10 for model details). The results revealed a significant Age Group effect for both current and future practice of social distancing, where the older group reported greater current practice of social distancing [*F*(1,676) = 16.16, *p* < 0.001, Cohen’s *d* = 0.31] and greater intention to practice social distancing in the future [*F*(1,676) = 9.22, *p* = 0.002, Cohen’s *d* = 0.23]. In addition, there was a significant spokesperson effect on current practice of social distancing, where the government official had a greater effect than the celebrity actor [*F*(1,676) = 4.84, *p* = 0.028, Cohen’s *d* = 0.17]. While the interaction of Age Group × Spokesperson was non-significant for current practice of social distancing, this effect, as can be seen in Figure 6, was larger in the older (Cohen’s *d* = 0.20) than the younger group (Cohen’s *d* = 0.11).

### Sensitivity Analysis

Since our sample was not representative of the Swiss general population, we performed Weighted Least Squares Regressions, wherein we weighted the study’s sample by the Swiss population demographic figures of 2019 for gender, age, and years of education (FSO, 2019). The weighting of these sample stratification variables was performed using the sequential weighting method, which allowed us to obtain unbiased estimates from the biased sample (Alkaya et al., 2017). First, we examined the effect of spokesperson and age on the social distancing measures, while also controlling for the study’s demographic and attitudinal factors. The regression model for current practice of social distancing measures showed that the weighted mean for the government official was higher than the celebrity actor, albeit at a non-significant level [*F*(1,676) = 3.15, *p* = 0.076, Cohen’s *d* = 0.14], and significantly lower among younger adults [*F*(1,676) = 21.69, *p* < 0.001, Cohen’s *d* = 0.36]. The regression model for future practice of social distancing measures showed that the weighted mean was significantly lower among younger adults [*F*(1,676) = 10.31, *p* = 0.001, Cohen’s *d* = 0.25], and similar for the government official and the celebrity actor (*p* = 0.784). There was no significant effect for either age (*p* = 0.322) or spokesperson (*p* = 0.675) on support for social distancing measures. In further analyses, taking into account the effect of likeability (Like vs. Neutral), the weighted means of the respondents’ current practice of social distancing measures was significantly higher for the government official [*F*(1,519) = 4.97, *p* = 0.026, Cohen’s *d* = 0.19] and lower among younger adults [*F*(1,519) = 4.97, *p* = 0.001, Cohen’s *d* = 0.29]; the effect of likeability was non-significant (*p* = 0.134) (see Supplementary Table 11 for model details). The effects of the spokesperson, age, and likeability on the respondents’ weighted means of support and future practice of social distancing measures were non-significant (*ps* > 0.073). These results largely consolidate our previous estimates obtained from the biased sample.

## Discussion

We discuss our results under three main headings: (1) the influence of spokesperson on compliance with social distancing measures; (2) respondents’ stance and attitudes toward social distancing measures; and (3) the association of demographic variables with compliance with social distancing measures.

### Spokesperson Influence

Social and physical distancing measures are paramount in preventing the spread of COVID-19 (Wilder-Smith and Freedman, 2020). Information about these measures has been communicated by various official and non-official sources. In an effort to provide evidence-base knowledge about who would be most effective in communicating recommended preventive health behavior, we tested if respondents were more likely to heed information conveyed by a government official or by a celebrity actor. Contrary to our prediction—namely, that the celebrity actor would be more effective than the government official due to a closer (perceived) relationship to the respondents (Basil, 1996)—the government official was in fact more effective, particularly with respect to the reported current compliance with social distancing measures (Figure 4). This effect was robust after adjusting for the effects of all demographic and attitudinal factors included in the study (Supplementary Table 3), and was largely confirmed in a sensitivity analysis in which we weighted our biased sample by the Swiss population demographic figures of 2019 for gender, age, and years of education (FSO, 2019). These results are consistent with previous studies showing that (1) a government official garners greater support and interest than a celebrity entertainer for *hypothetical* crises (Frizzell, 2011). During times of crises, people tend to rally around their leaders in the hope for assurance. Indeed, it has been well-documented that government leaders tend to elicit higher approval and trust ratings during times of crises (Gaines, 2002; Gregg, 2003). Furthermore, although there was a general trend for greater endorsement of the social distancing measures among those who liked the spokesperson, this was non-significant (Figure 5). This suggests that the likeability of a government leader may largely be insubstantial in the development of strategies for improving the adoption of measures for social distancing, since it is a factor that cannot be easily adjusted—exchanging an (elected) government official is typically not an option.

### Stance and Attitudinal Variables Toward Social Distancing Measures

Concerning the relationship between risk perception, and attitudinal variables with the stance toward social distancing measures, we highlight key results. First, respondents who indicated greater support, and current and future practice of social distancing measures also expressed (1) higher concern for the current situation, (2) higher concern for the well-being of others, (3) higher belief that others are practicing social distancing, and (4) lower perceived mobility (see Figure 2 and Supplementary Tables 2–4). The association between social distancing and the concern for others is consistent with the results of a German survey, which showed that this association was particularly strong when the motivation was to protect the vulnerable (Betsch, 2020). This association can be interpreted from a pro-sociality point of view. While compliance can be seen as a response to protect oneself, it may also be motivated by the desire to protect others. In this regard, recent research has demonstrated that inducing empathy for people most vulnerable to the coronavirus promotes the motivation to adhere to these measures (Pfattheicher et al., 2020), which is consistent, the authors point out, with research suggesting that the motivation to adhere to social distancing measures includes concerns for both self and others (Wise et al., 2020). In addition, the association of perceived mobility with social distancing has also been recognized as an important variable in the development of messages and policies that are most effective. Specifically, it has been suggested that examining the impact of social distancing messaging on population mobility patterns, will help officials understand what kinds of messaging are most effective (Buckee et al., 2020). Collectively, these results can be interpreted in terms of the PMT (Maddux and Rogers, 1983), which, as stated in the introduction, attempts to explain the effects of threatening health information on attitude and behavior change in terms of threat appraisal and coping appraisal processes. In the context of our results, concern for the situation, others’ practice of social distancing, and mobility can be construed as part of the threat appraisal process, while concern for others can be construed as part of the coping appraisal process, which can manifest by empathizing and helping the vulnerable (Betsch, 2020).

In addition, when taking demographic and other attitudinal measures into account, we found that individuals who reported greater importance for religion in their daily life also expressed less support for the social distancing measures (Supplementary Tables 2, 5). This result is contrary to the findings by Everett et al. (2020) who found a positive association between religiosity and adherence to social distancing measures among American respondents. It is possible that the negative association we observe is reflective of the notion that more religious people have a preference for persistence and consistency over flexibility and change (Zmigrod et al., 2019). Finally, respondent’s general health, perception of spread of the disease, satisfaction with the government’s efforts to combat COVID-19, and perception of the government’s concern for public health versus the economy were of little importance in predicting engagement in social distancing.

### Demographic Variables

As discussed above (see section “Spokesperson Influence”), we found evidence suggesting that the government official was more effective than the celebrity spokesperson in communicating recommended preventive health behavior. It appears that this effect is stronger among older respondents (Figure 6). Intriguingly, however, we observed that support and reported compliance was higher among older versus younger respondents, despite older respondents having lower risk perception as indicated by their own assessment of the spread of COVID-19 (Figure 3). This result is consistent with the findings of COVID-19 research showing that younger respondents exhibit attenuated support of, and compliance with social distancing measures (Barari et al., 2020; Everett et al., 2020), and that older people have lower risk perception (Betsch, 2020). Importantly, our finding consolidate previous conclusions drawn from previous pandemics, such as the 2009 H1NA pandemic (Bish and Michie, 2010), suggesting that being older was associated with a better chance of adopting behaviors that could contribute to controlling the spread of pandemic disease.

In addition, we found that support and current practice of social distancing were inversely related with settlement size (Supplementary Tables 2, 3). This dovetails with the findings of a recent study showing that the spread of COVID-19 in the United States increases with city size (Stier et al., 2020), and suggests that different communication strategies for social distancing in rural versus urban settings (Vaughan and Tinker, 2009) may be needed, not least because both settings are clearly governed by markedly different socioeconomic interactions (Stier et al., 2020). We also found evidence suggesting that household size is inversely associated with compliance with social distancing measures (see Figure 2 and Supplementary Tables 9, 11), and that current practice of social distancing was significantly lower among the employed (see Supplementary Tables 3, 11). Finally, respondent’s level of education and gender status were of little importance in predicting engagement in social distancing.

### Strengths and Limitations

The study’s results contribute to the development of evidence-based knowledge regarding the influence of the spokesperson on the effectiveness of public health messaging during times of emerging infectious diseases; the results were obtained while controlling for a number of relevant demographic, attitudinal and psychological factors, and which were largely confirmed in a sensitivity analysis adjusting for the representativeness of the study sample in terms of the Swiss population demographic figures for gender, age, and education (FSO, 2019). However, given the complexity of the issue and the experimental design, this study has a number of limitations that we discuss in the following.

#### Time Frame

The data were necessarily collected within a short period of time, due to the highly dynamic nature of COVID-19 and the continuous introduction of new social and physical distancing measures. These conditions may have affected respondents differently depending on the time at which they completed the survey. Furthermore, this time frame is not representative for the entire duration of the pandemic, and a survey of longitudinal effects could be useful in determining long-term adoption and acceptance of measures. However, obtaining data during this timeframe of the COVID-19 pandemic may be particularly informative about the effectiveness of the spokesperson during the early stages of emerging infectious diseases, which could greatly affect how individuals interpret health risk communications throughout the course of the pandemic.

#### Experimental Biases

As with all online surveys, our analyses are based on self-reported measures that might be susceptible to confirmation bias. Furthermore, our main outcome measures suffered from ceiling effects (but note that we nonetheless observed significant effects) that should be addressed in future experiments.

#### Spokespersons

We only compared two spokespersons in this study, a government official and an international celebrity who had been infected and outspoken about the pandemic prior to the survey. In the context of COVID-19, future research should consider a more diverse set of parameters for the selection of spokespersons, for example by including a scientist spokesperson or a citizen spokesperson, to better gauge the effectiveness of celebrity and government officials in communicating preventive health recommendations, particularly in the early stages of the pandemic when many of the facts may be uncertain. For celebrity spokespersons, it would also be pertinent to consider celebrities from different domains (e.g., the music or film industry, athletes, or even celebrities in science and education). Finally, the effect of the spokespersons’ gender, ethnicity, and nationality on the reported adherence to distancing measures should be considered.

#### Political and Cultural Differences

For our study, we focused on Switzerland. However, one may reasonably expect differences in actual or reported behavior of people from different cultural background or political systems, e.g., in countries where free speech is not guaranteed and respondents may have to fear repercussions for perceived disobedience to authority.

#### Likeability

Our ability to gauge the effect of likeability was limited to a “like” versus “neutral” attitude toward the spokesperson. Perhaps a continuous measure would provide more testing power, and a larger sample size and more diverse selection of spokespersons might provide results for the response to “disliked” spokespersons.

#### Sample and Representativeness

Our sample size is relatively small and thus our ability to detect additional significant effects might be hampered by lack of power. In addition, our sample was not representative of the Swiss general population. It consisted of university students and Facebook users, who were highly educated (80% with ≥14 years of education), younger adults (60% between 18 and 34 years of age), and mostly females (78%). However, the sensitivity analysis suggests that the effect of age may be generalizable to the general Swiss population and that the spokesperson effect may be worthy of further investigation in subsequent, more highly powered studies of representative samples.

#### Self-Reported Versus Actual Behavior

Since our data solely consisted of self-reports, the extent to which observed effects reflect actual, rather than merely reported social distancing behavior is unknown. We emphasize, however, that our findings are nonetheless consistent with previous research on actual COVID-19 related behavior (Buckee et al., 2020), and that self-reported social distancing measures seem to reflect real-world behavior (Gollwitzer et al., 2020).

## Conclusion

Even with the availability of a vaccine and improved medical treatment, strict social and physical distancing measures are necessary and perhaps our best strategy in combating the spread of COVID-19, which may need to be sustained as late as 2022 (Kissler et al., 2020). However, ensuring that these measures are enforceable for an extended period of time will be challenging. The limitations of our study notwithstanding, and consistent with lessons drawn from past pandemics (Vaughan and Tinker, 2009; Bish and Michie, 2010; Lyu et al., 2013), we can offer a number of recommendations that may help face these challenges. Our findings suggest that having an effective spokesperson might further increase adherence to these measures. Importantly, however, since different parts of the population appear to have different perceptions of risk and crisis, our findings also suggest that different spokespersons may be needed for different segments of the population and particularly for younger versus older populations. Evidence-based knowledge is thus required to further identify who would be the most effective spokesperson, in particular to groups with low risk perception and low compliance. While the effect sizes of our study are small, in the context of the COVID-19 pandemic, a modest effect can translate into saving the lives of thousands. Furthermore, the applicability of our findings is not limited to the COVID-19 pandemic, since they stand to be useful in the context of other respiratory infections, for which similar social distancing measures have been proposed (Glass et al., 2006; Bish and Michie, 2010; Qualls et al., 2017). Collectively, these findings may provide practical insight for the development of strategies to help mitigate this as well as future impending crises, and suggest that while previous research on the communication efficacy of public health messaging during pandemics reflect thoughtful, evidence-based strategies, they could be strengthened by having more emphasis on the messenger and not just the message.

## Data Availability Statement

The raw data supporting the conclusions of this article will be made available by the authors, without undue reservation.

## Ethics Statement

The studies involving human participants were reviewed and approved by the EPFL Human Research Ethics Committee. The patients/participants provided their written informed consent to participate in this study.

## Author Contributions

AA-A, AS, and RW contributed to the conceptualization of the study, design, and the administration of the survey. AA-A performed the analysis and drafted the manuscript. AS and RW provided statistical suggestion on the data analysis and contributed to the writing of the manuscript. All authors reviewed and approved the final version of the manuscript.

## Conflict of Interest

The authors declare that the research was conducted in the absence of any commercial or financial relationships that could be construed as a potential conflict of interest.
