# Supplementary material for: Who Is Listening? Spokesperson Effect on Communicating Social and Physical Distancing Measures During the COVID-19 Pandemic
Source: Front Psychol. 2021 Jan 12;11:564434. doi: 10.3389/fpsyg.2020.564434 (PMC7837291; doi:10.3389/fpsyg.2020.564434)
Supplement: Supplementary file 1 [file Data_Sheet_1.PDF]

Supplementary Material for

Who is Listening? Spokesperson Effect on Communicating Social and Physical Distancing Measures During the COVID-19 Pandemic

Ahmad Abu-Akel[1*§], Andreas Spitz[2§], Robert West[2*]

[1] Institute of Psychology, University of Lausanne,

Lausanne, Switzerland

[2] Institute of Computer and Communication Sciences,

École Polytechnique Fédérale de Lausanne, Lausanne, Switzerland

[*] Correspondence to: ahmad.abuakel@unil.ch *or* robert.west@epfl.ch
[§] AAA and AS contributed equally to this work.

1. Effect of age on social distancing and attitudinal measures

Age distribution and clustering: Due to the non-unimodal structure of the age distribution of our sample (Hartigans' dip test (D_{5000}) = 0.05, p < 2.2e-16), we performed a 2-step cluster analysis, using Schwarz's Bayesian Criterion, to identify potential subgroups. A two-cluster solution was deemed optimal with a silhouette score of 0.7 (a measure of "cohesion and separation" of clusters) (Rousseeuw, 1987), suggesting a good cluster structure (see Fig. S1): A young group (N =445, M/F = 89/336, Mean age ± σ = 22.32 ± 3.92, Range = 17-36 years), and an older group (N=280, M/F= 68/212, Mean age ± σ = 52.60 ± 10.25, Range = 37-80 years).

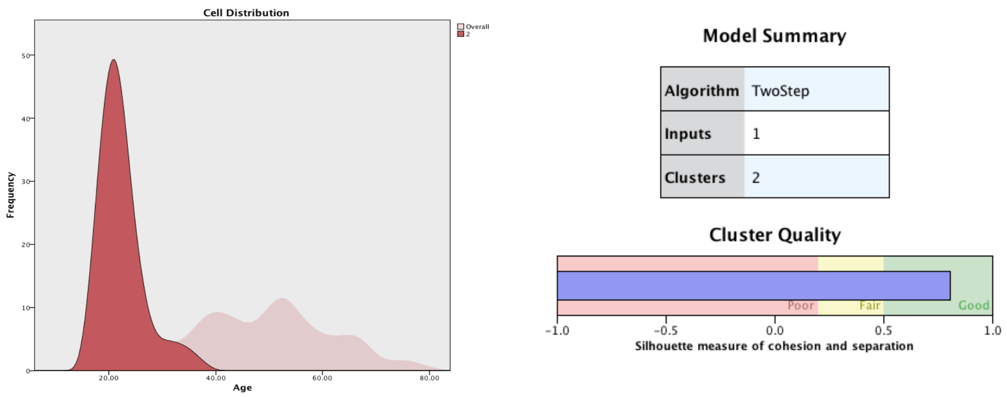

Fig. S1. Density plot of the age distribution and the result of the two-step cluster analysis

Table S1. Differences between the young and old age groups on social distancing and attitudinal measures

Measure	Young (N = 425)		Old (N =280)		Test	p-value		Effect size
	Mean	SD	Mean	SD	KW H*	Un-corrected	FDR corrected	Cohen's d
Support for social distancing	6.38	0.99	6.61	0.93	18.724	0.000	**0.000**	0.24
Current practice of social distancing	6.04	1.06	6.56	0.82	68.303	0.000	**0.000**	0.56
Future practice of future distancing	6.03	1.07	6.48	0.93	47.716	0.000	**0.000**	0.45
Concern for the situation	4.78	1.36	5.64	1.26	67.184	0.000	**0.000**	0.65
Concern for others	5.64	1.23	6.26	1.03	54.238	0.000	**0.000**	0.54
Others' practice of social distancing	4.07	1.06	4.52	1.02	27.575	0.000	**0.000**	0.43
Religiosity	2.20	1.76	2.83	1.95	21.539	0.000	**0.000**	0.34
Liberty of Movement (Mobility)	1.59	1.11	1.63	1.43	4.458	0.043	*0.052*	0.03
Satisfaction from government	4.06	1.64	3.93	1.87	0.913	0.300	0.327	0.08
Government prioritizing public health over economy	3.38	1.73	3.45	1.94	0.090	0.709	0.709	0.04
Percent spread of COVID-19	2.89	1.87	2.58	1.78	5.770	0.012	**0.018**	0.17
Subjective health	4.01	0.82	3.84	0.89	5.464	0.024	**0.032**	0.20

* KW = Kruskal-Wallis H test; SD = Standard Deviation

1. Effect of spokesperson on endorsing social distancing measures

Table S2. Parameter estimates of the multivariable regression for support of social distancing

	**Model**<br>F(16,688)= 8.264, $p< .001$, $\eta_p^2 = .161$	**B**	**SE**	**t**	**Sig.**	**95% CI**		η_p^2
**Support for Social Distancing**	Intercept	4.346	0.387	11.239	**0.000**	3.587	5.105	0.155
	Age	0.003	0.002	1.344	0.179	-0.002	0.008	0.003
	Gender=Female	0.108	0.084	1.295	0.196	-0.056	0.272	0.002
	Employment status= Employed	-0.054	0.071	-0.764	0.445	-0.193	0.085	0.001
	Years of Education	0.017	0.035	0.484	0.629	-0.052	0.086	0.000
	Settlement size	-0.070	0.033	-2.125	**0.034**	-0.135	-0.005	0.007
	Household size	-0.018	0.027	-0.661	0.509	-0.071	0.035	0.001
	Concern for the situation	0.087	0.031	2.839	**0.005**	0.027	0.147	0.012
	Concern for others	0.131	0.033	3.944	**0.000**	0.066	0.197	0.022
	Others' practice of social distancing	0.144	0.035	4.075	**0.000**	0.075	0.214	0.024
	Religiosity	-0.064	0.019	-3.384	**0.001**	-0.101	-0.027	0.016
	Liberty of Movement (Mobility)	-0.136	0.028	-4.837	**0.000**	-0.191	-0.081	0.033
	Satisfaction from government	0.047	0.025	1.897	*0.058*	-0.002	0.095	0.005
	Government prioritizing public health over economy	0.026	0.022	1.181	0.238	-0.017	0.070	0.002
	Percent spread of COVID-19	0.029	0.020	1.469	0.142	-0.010	0.068	0.003
	Subjective health	0.045	0.042	1.076	0.282	-0.037	0.126	0.002
	Spokesperson=Government	0.022	0.069	0.324	0.746	-0.114	0.159	0.000

Table S3. Parameter estimates of the multivariable regression for current practice of social distancing

	**Model**<br>F(16,688)= 15.299, $p< .001$, $\eta_p^2 = .262$	**B**	**SE**	**t**	**Sig.**	**95% CI**		η_p^2
**Current Practice of Social Distancing**	Intercept	4.251	0.374	11.356	**0.000**	3.516	4.985	0.158
	Age	0.010	0.002	4.197	**0.000**	0.005	0.015	0.025
	Gender=Female	0.035	0.081	0.439	0.661	-0.123	0.194	0.000
	Employment status= Employed	-0.213	0.069	-3.112	**0.002**	-0.348	-0.079	0.014
	Years of Education	0.014	0.034	0.409	0.683	-0.053	0.081	0.000
	Settlement size	-0.102	0.032	-3.177	**0.002**	-0.165	-0.039	0.014
	Household size	-0.049	0.026	-1.849	0.065	-0.100	0.003	0.005
	Concern for the situation	0.133	0.030	4.490	**0.000**	0.075	0.191	0.028
	Concern for others	0.158	0.032	4.908	**0.000**	0.095	0.221	0.034
	Others' practice of social distancing	0.108	0.034	3.140	**0.002**	0.040	0.175	0.014
	Religiosity	-0.035	0.018	-1.892	*0.059*	-0.070	0.001	0.005
	Liberty of Movement (Mobility)	-0.171	0.027	-6.292	**0.000**	-0.225	-0.118	0.054
	Satisfaction from government	0.028	0.024	1.153	**0.000**	-0.019	0.074	0.002
	Government prioritizing public health over economy	0.005	0.022	0.216	0.829	-0.038	0.047	0.000
	Percent spread of COVID-19	0.027	0.019	1.418	0.157	-0.011	0.065	0.003
	Subjective health	0.010	0.040	0.260	0.795	-0.069	0.089	0.000
	Spokesperson=Government	0.151	0.067	2.251	**0.025**	0.019	0.283	0.007

Table S4. Parameter estimates of the multivariable regression for future practice of social distancing

	**Model**<br>F(16,688)= 10.158, p< .001, $\boldsymbol{\eta_p^2}$ = .191	**B**	**SE**	**t**	**Sig.**	**95% CI**		$\boldsymbol{\eta_p^2}$
**Future Practice of Social Distancing**	Intercept	4.094	0.406	10.084	**0.000**	3.297	4.891	0.129
	Age	0.007	0.003	2.726	**0.000**	0.002	0.012	0.011
	Gender=Female	0.021	0.088	0.235	0.814	-0.152	0.193	0.000
	Employment status= Employed	-0.009	0.074	-0.128	0.898	-0.155	0.136	0.000
	Years of Education	0.006	0.037	0.152	0.879	-0.067	0.078	0.000
	Settlement size	-0.059	0.035	-1.713	*0.087*	-0.128	0.009	0.004
	Household size	0.016	0.029	0.555	0.579	-0.040	0.072	0.000
	Concern for the situation	0.090	0.032	2.800	**0.005**	0.027	0.153	0.011
	Concern for others	0.177	0.035	5.078	**0.000**	0.109	0.246	0.036
	Others' practice of social distancing	0.125	0.037	3.371	**0.001**	0.052	0.198	0.016
	Religiosity	-0.026	0.020	-1.312	0.190	-0.065	0.013	0.002
	Liberty of Movement (Mobility)	-0.182	0.030	-6.151	**0.000**	-0.240	-0.124	0.052
	Satisfaction from government	0.036	0.026	1.370	0.171	-0.015	0.086	0.003
	Government prioritizing public health over economy	0.008	0.023	0.327	0.744	-0.038	0.054	0.000
	Percent spread of COVID-19	0.005	0.021	0.249	0.803	-0.036	0.046	0.000
	Subjective health	0.000	0.044	-0.011	0.991	-0.086	0.085	0.000
	Spokesperson=Government	0.077	0.073	1.060	0.290	-0.066	0.220	0.002

2. Effect of spokesperson's likeability on endorsing social distancing measures

Table S5. Parameter estimates of the multivariable regression for support of social distancing

	**Model**<br>$F(18,527)= 5.553$, $p< .001$, $\eta_p^2 = .159$	**B**	**SE**	**t**	**Sig.**	**95% CI**		η_p^2
**Support for Social Distancing**	Intercept	4.782	0.428	11.179	**0.000**	3.941	5.622	0.192
	Age	0.002	0.003	0.646	0.519	-0.004	0.007	0.001
	Gender=Female	0.100	0.090	1.106	0.269	-0.078	0.277	0.002
	Employment status= Employed	-0.077	0.077	-0.997	0.319	-0.228	0.075	0.002
	Years of Education	0.019	0.038	0.511	0.610	-0.055	0.094	0.000
	Settlement size	-0.073	0.036	-1.993	**0.047**	-0.144	-0.001	0.007
	Household size	-0.027	0.029	-0.936	0.350	-0.084	0.030	0.002
	Concern for the situation	0.083	0.033	2.509	**0.012**	0.018	0.149	0.012
	Concern for others	0.091	0.036	2.490	**0.013**	0.019	0.162	0.012
	Others' practice of social distancing	0.166	0.040	4.161	**0.000**	0.088	0.244	0.032
	Religiosity	-0.062	0.021	-2.981	**0.003**	-0.102	-0.021	0.017
	Liberty of Movement (Mobility)	-0.156	0.033	-4.727	**0.000**	-0.221	-0.091	0.041
	Satisfaction from government	0.024	0.027	0.879	0.380	-0.029	0.077	0.001
	Government prioritizing public health over economy	0.024	0.024	0.979	0.328	-0.024	0.071	0.002
	Percent spread of COVID-19	0.022	0.021	1.048	0.295	-0.020	0.065	0.002
	Subjective health	0.036	0.045	0.798	0.425	-0.053	0.125	0.001
	Spokesperson=Government	0.013	0.099	0.136	0.892	-0.181	0.207	0.000
	Likeability=Like	0.058	0.102	0.565	0.572	-0.142	0.258	0.001
	Spokesperson x Likeability	0.105	0.151	0.692	0.489	-0.193	0.402	0.001

Table S6. Parameter estimates of the multivariable regression for current practice of social distancing

**Model** $F(18,527)= 8.191, p< .001, \eta_p^2 = .197$		**B**	**SE**	**t**	**Sig.**	**95% CI**		η_p^2
**Current Practice of Social Distancing**	Intercept	4.644	0.429	10.835	**0.000**	3.802	5.486	0.182
	Age	0.010	0.003	3.532	**0.000**	0.004	0.015	0.023
	Gender=Female	-0.037	0.091	-0.410	0.682	-0.215	0.141	0.000
	Employment status= Employed	-0.253	0.077	-3.272	**0.001**	-0.404	-0.101	0.020
	Years of Education	0.021	0.038	0.545	0.586	-0.054	0.096	0.001
	Settlement size	-0.109	0.037	-2.989	**0.003**	-0.181	-0.037	0.017
	Household size	-0.038	0.029	-1.290	0.198	-0.095	0.020	0.003
	Concern for the situation	0.130	0.033	3.898	**0.000**	0.064	0.195	0.028
	Concern for others	0.120	0.036	3.287	**0.001**	0.048	0.191	0.020
	Others' practice of social distancing	0.110	0.040	2.751	**0.006**	0.031	0.189	0.014
	Religiosity	-0.026	0.021	-1.255	0.210	-0.067	0.015	0.003
	Liberty of Movement (Mobility)	-0.147	0.033	-4.441	**0.000**	-0.212	-0.082	0.036
	Satisfaction from government	0.020	0.027	0.747	0.455	-0.033	0.073	0.001
	Government prioritizing public health over economy	0.002	0.024	0.083	0.934	-0.046	0.050	0.000
	Percent spread of COVID-19	0.007	0.022	0.331	0.741	-0.035	0.049	0.000
	Subjective health	0.019	0.045	0.410	0.682	-0.071	0.108	0.000
	Spokesperson=Government	0.112	0.099	1.134	0.257	-0.082	0.307	0.002
	Likeability=Like	-0.011	0.102	-0.109	0.913	-0.212	0.189	0.000
	Spokesperson x Likeability	0.063	0.152	0.418	0.676	-0.235	0.362	0.000

Table S7. Parameter estimates of the multivariable regression for future practice of social distancing

	**Model**<br>F(18,527)= 7.182, p< .001, η_p^2 = .191	**B**	**SE**	**t**	**Sig.**	**95% CI**		η_p^2
**Future Practice of Social Distancing**	Intercept	4.322	0.456	9.479	**0.000**	3.426	5.218	0.146
	Age	0.006	0.003	2.219	**0.027**	0.001	0.012	0.009
	Gender=Female	-0.033	0.096	-0.346	0.730	-0.222	0.156	0.000
	Employment status= Employed	0.020	0.082	0.247	0.805	-0.141	0.182	0.000
	Years of Education	0.013	0.041	0.330	0.742	-0.066	0.093	0.000
	Settlement size	-0.078	0.039	-2.010	**0.045**	-0.155	-0.002	0.008
	Household size	0.017	0.031	0.538	0.591	-0.044	0.078	0.001
	Concern for the situation	0.118	0.035	3.325	**0.001**	0.048	0.188	0.021
	Concern for others	0.135	0.039	3.470	**0.001**	0.058	0.211	0.022
	Others' practice of social distancing	0.116	0.043	2.716	**0.007**	0.032	0.199	0.014
	Religiosity	-0.039	0.022	-1.774	*0.077*	-0.083	0.004	0.006
	Liberty of Movement (Mobility)	-0.196	0.035	-5.572	**0.000**	-0.265	-0.127	0.056
	Satisfaction from government	0.023	0.029	0.796	0.426	-0.034	0.080	0.001
	Government prioritizing public health over economy	-0.007	0.026	-0.287	0.774	-0.058	0.043	0.000
	Percent spread of COVID-19	0.005	0.023	0.202	0.840	-0.040	0.050	0.000
	Subjective health	0.034	0.048	0.703	0.482	-0.061	0.129	0.001
	Spokesperson=Government	0.200	0.105	1.897	*0.058*	-0.007	0.407	0.007
	Likeability=Like	0.166	0.109	1.528	0.127	-0.047	0.379	0.004
	Spokesperson x Likeability	-0.101	0.161	-0.627	0.531	-0.418	0.216	0.001

3. Effect of spokesperson in younger and older adults

Table S8. Parameter estimates of the multivariable regression for support of social distancing

	**Model**<br>F(17,676)= 7.666, p< .001, η_p^2 = .162	**B**	**SE**	**t**	**Sig.**	**95% CI**		η_p^2
**Support for Social Distancing**	Intercept	4.494	0.402	11.178	**0.000**	3.704	5.283	0.156
	Gender=Female	0.105	0.085	1.231	0.219	-0.062	0.272	0.002
	Employment status= Employed	-0.055	0.073	-0.753	0.452	-0.198	0.088	0.001
	Years of Education	0.021	0.036	0.577	0.564	-0.050	0.091	0.000
	Settlement size	-0.079	0.035	-2.289	**0.022**	-0.147	-0.011	0.008
	Household size	-0.024	0.026	-0.890	0.374	-0.075	0.028	0.001
	Concern for the situation	0.088	0.031	2.851	**0.004**	0.027	0.149	0.012
	Concern for others	0.130	0.034	3.844	**0.000**	0.064	0.197	0.021
	Others' practice of social distancing	0.150	0.036	4.124	**0.000**	0.079	0.221	0.025
	Religiosity	-0.058	0.019	-3.034	**0.003**	-0.095	-0.020	0.013
	Liberty of Movement (Mobility)	-0.136	0.028	-4.789	**0.000**	-0.192	-0.080	0.033
	Satisfaction from government	0.049	0.025	1.937	*0.053*	-0.001	0.098	0.006
	Government prioritizing public health over economy	0.026	0.023	1.162	0.246	-0.018	0.071	0.002
	Percent spread of COVID-19	0.030	0.020	1.479	0.140	-0.010	0.070	0.003
	Subjective health	0.051	0.043	1.209	0.227	-0.032	0.135	0.002
	Spokesperson=Government	-0.043	0.111	-0.388	0.698	-0.261	0.175	0.000
	Age Group=Younger Adults	-0.141	0.110	-1.282	0.200	-0.358	0.075	0.002
	Spokesperson x Age Group	0.115	0.142	0.807	0.420	-0.165	0.394	0.001

Table S9. Parameter estimates of the multivariable regression for current practice of social distancing

	$F(17,676) = 14.292$, $p < .001$, $\eta_p^2 = .264$	B	SE	t	Sig.	95% CI		η_p^2
**Current Practice of Social Distancing**	Intercept	4.868	0.387	12.571	**0.000**	4.107	5.628	0.189
	Gender=Female	0.010	0.082	0.124	0.902	-0.151	0.171	0.000
	Employment status= Employed	-0.245	0.070	-3.500	**0.000**	-0.383	-0.108	0.018
	Years of Education	0.015	0.035	0.441	0.659	-0.053	0.083	0.000
	Settlement size	-0.120	0.033	-3.608	**0.000**	-0.186	-0.055	0.019
	Household size	-0.063	0.025	-2.456	**0.014**	-0.112	-0.013	0.009
	Concern for the situation	0.134	0.030	4.488	**0.000**	0.075	0.192	0.029
	Concern for others	0.159	0.033	4.861	**0.000**	0.095	0.223	0.034
	Others' practice of social distancing	0.111	0.035	3.156	**0.002**	0.042	0.179	0.015
	Religiosity	-0.028	0.018	-1.510	0.132	-0.064	0.008	0.003
	Liberty of Movement (Mobility)	-0.172	0.027	-6.285	**0.000**	-0.226	-0.118	0.055
	Satisfaction from government	0.025	0.024	1.046	0.296	-0.022	0.073	0.002
	Government prioritizing public health over economy	0.005	0.022	0.248	0.804	-0.037	0.048	0.000
	Percent spread of COVID-19	0.027	0.019	1.364	0.173	-0.012	0.065	0.003
	Subjective health	0.012	0.041	0.295	0.768	-0.068	0.093	0.000
	Spokesperson=Government	0.198	0.107	1.854	*0.064*	-0.012	0.407	0.005
	Age Group=Younger Adults	-0.275	0.106	-2.590	**0.010**	-0.483	-0.066	0.010
	Spokesperson x Age Group	-0.091	0.137	-0.663	0.507	-0.360	0.178	0.001

Table S10. Parameter estimates of the multivariable regression for future practice of social distancing

	**Model**<br>F(17,676)= 9.512, p< .001, η_p^2 = .193	**B**	**SE**	**t**	**Sig.**	**95% CI**		η_p^2
**Future Practice of Social Distancing**	Intercept	4.497	0.421	10.681	**0.000**	3.671	5.324	0.144
	Gender=Female	0.019	0.089	0.218	0.828	-0.156	0.195	0.000
	Employment status= Employed	-0.029	0.076	-0.387	0.699	-0.179	0.120	0.000
	Years of Education	0.012	0.038	0.329	0.742	-0.062	0.086	0.000
	Settlement size	-0.063	0.036	-1.744	*0.082*	-0.134	0.008	0.004
	Household size	0.010	0.028	0.346	0.729	-0.045	0.064	0.000
	Concern for the situation	0.087	0.032	2.677	**0.008**	0.023	0.151	0.010
	Concern for others	0.172	0.036	4.846	**0.000**	0.102	0.242	0.034
	Others' practice of social distancing	0.125	0.038	3.281	**0.001**	0.050	0.200	0.016
	Religiosity	-0.024	0.020	-1.182	0.238	-0.063	0.016	0.002
	Liberty of Movement (Mobility)	-0.184	0.030	-6.183	**0.000**	-0.242	-0.126	0.054
	Satisfaction from government	0.038	0.026	1.442	0.150	-0.014	0.089	0.003
	Government prioritizing public health over economy	0.009	0.024	0.400	0.689	-0.037	0.056	0.000
	Percent spread of COVID-19	0.006	0.021	0.305	0.760	-0.035	0.048	0.000
	Subjective health	0.006	0.045	0.134	0.894	-0.082	0.093	0.000
	Spokesperson=Government	0.107	0.116	0.923	0.356	-0.121	0.335	0.001
	Age Group= Younger Adults	-0.244	0.115	-2.116	**0.035**	-0.471	-0.018	0.007
	Spokesperson x Age Group	-0.038	0.149	-0.253	0.800	-0.330	0.255	0.000

Table S11. Parameter estimates of the multivariable weighted regression for current practice of social distancing

	**Model** F(17, 519)= 10.718, p< .001, η_p^2 = .260	**B**	**SE**	**t**	**Sig.**	**95% CI**		η_p^2
**Current Practice of Social Distancing**	Intercept	4.678	0.364	12.846	0.000	3.963	5.393	0.241
	Age Group= Younger Adults	-0.271	0.081	-3.362	**0.001**	-0.43	-0.113	0.021
	Gender=Female	0.029	0.073	0.396	0.692	-0.115	0.173	0.000
	Employment status= Employed	-0.268	0.073	-3.653	**0.000**	-0.412	-0.124	0.025
	Years of Education	0.03	0.031	0.959	0.338	-0.032	0.092	0.002
	City size	-0.119	0.037	-3.21	**0.001**	-0.192	-0.046	0.019
	Household size	-0.055	0.021	-2.669	**0.008**	-0.095	-0.014	0.014
	Concern for the situation	0.109	0.031	3.517	**0.000**	0.048	0.169	0.023
	Concern for others	0.164	0.034	4.773	**0.000**	0.097	0.232	0.042
	Others practice of social distancing	0.122	0.038	3.168	**0.002**	0.046	0.198	0.019
	Religiosity	-0.012	0.019	-0.629	0.529	-0.05	0.026	0.001
	Liberty of Movement (Mobility)	-0.089	0.029	-3.025	**0.003**	-0.146	-0.031	0.017
	Satisfaction from government	0.019	0.026	0.745	0.456	-0.031	0.069	0.001
	Government prioritizing public health over economy	0.005	0.022	0.228	0.819	-0.038	0.048	0.000
	Percent spread of COVID-19	-0.015	0.021	-0.722	0.471	-0.056	0.026	0.001
	General health	0.08	0.043	1.88	0.061	-0.004	0.164	0.007
	Spokesperson=Government	0.162	0.073	2.228	**0.026**	0.019	0.304	0.009
	Likeability=Like	-0.113	0.075	-1.508	0.132	-0.26	0.034	0.004

References

1. P. J. Rousseeuw, Silhouettes: a graphical aid to the interpretation and validation of cluster analysis. *Journal of computational and applied mathematics* 20, 53-65 (1987).

Appendix: Survey*

If you have 2-3 minutes, we would greatly appreciate it if you could take this short survey to tell us how the spread of coronavirus disease (COVID-19) is affecting your life. We are a university research lab trying to better understand how people are dealing with the crisis. Your input matters!

This survey is anonymous.

**Q1**	How worried are you about the COVID-19 situation in Switzerland right now?	(1) I am not worried<br>(7) I am extremely worried
**Q2**	Please share your opinion about the response of the Swiss government and of the Swiss population to COVID-19.	Text input

In an effort to avoid spreading COVID-19, a commonly given instruction is to practice SOCIAL DISTANCING, that is, to deliberately stay away from other people by at least 2 meters (6 feet).

Examples of social distancing are
- canceling sports events, cruises, festivals and other gatherings,
- working from home instead of at the office,
- closing schools and universities or switching to online classes,
- visiting loved ones by electronic devices instead of in person,
- canceling or postponing conferences and large meetings.

<Image of spokesperson>

Social distancing has been publicly supported, among others, by <spokesperson>.

**Q3**	Were you aware of the instruction to practice social distancing?	Yes / No
**Q4**	To what degree do you support social distancing as a valid measure in the current situation?	(1) I don't support it<br>(7) I fully support it
**Q5**	To what degree are you currently practicing social distancing?	(1) Not at all<br>(7) All the time
**Q6**	To what degree do you think others are currently practicing social distancing?	(1) Not at all<br>(7) All the time
**Q7**	To what degree do you see yourself practicing social distancing in the weeks to come?	(1) Not at all<br>(7) All the time
**Q8**	How do you feel about <speaker>?	I like <speaker><br>I neither like nor dislike <speaker><br>I dislike <speaker><br>(I don't know <speaker>)
**Q9**	What is your personal estimate of the percentage of people in your place of residence (city/town/village) who are actually already infected by coronavirus? (Give your best personal guess of the percentage of *actually* infected people (tested + untested), not the official statistics of people who tested positive.)	0-9%<br>10-19%<br>...<br>90-100%
**Q10**	How concerned are you for the well-being of your fellow citizens at the current time?	(1) Not at all<br>(7) Very concerned
**Q11**	How would you rate your overall health in the last 30 days?	Very good<br>Good<br>Average<br>Bad<br>Very bad

Considering the current situation in Switzerland, please state the level to which you agree with the following statements.

**Q12**	"I feel free to move around and travel wherever I need to in order to go about my daily life, to attend appointments or to visit family or friends."	(1) Disagree strongly<br>(7) Agree strongly
**Q13**	"I am satisfied with the Swiss government's effort and preparedness to fight COVID-19."	(1) Disagree strongly<br>(7) Agree strongly
**Q14**	"I think the Swiss government cares more about public health than about the economy."	(1) Disagree strongly<br>(7) Agree strongly

Please share some details about yourself.

**Q15**	Gender	Female / Male / Other
**Q16**	Age	Numerical input
**Q17**	How many years (full-time equivalent) have you been in formal education? Include all primary and secondary schooling, university and other post-secondary education, and full-time vocational training, but do not include repeated years. If you are currently in education, count the number of years you have completed so far.	I have no formal schooling<br>1-6 years<br>7-13 years<br>14-16 years<br>17-18 years<br>More than 18 years
**Q18**	Are you currently employed?	Yes / No
**Q19**	What is your current country of residence?	Text input
**Q20**	Which of the following best describes the area in which you live?	Village / rural area (fewer than 3,000 people)<br>Small town (3,000 to 15,000 people)<br>Town (15,000 to 100,000 people)<br>City (100,000 to 1,000,000 people)<br>Metropolitan area (over 1,000,000 people)
**Q21**	How many people live in your household or shared apartment (including you)?	Numerical input
**Q22**	How important is religion in your daily life?	(1) Not important at all<br>(7) Very important

* Original survey was administered in French
